# Effect of PEPFAR funding policy change on HIV service delivery in a large HIV care and treatment network in Nigeria

**DOI:** 10.1371/journal.pone.0221809

**Published:** 2019-09-25

**Authors:** Bolanle Banigbe, Carolyn M. Audet, Prosper Okonkwo, Olujide O. Arije, Elizabeth Bassi, Kate Clouse, Melynda Simmons, Muktar H. Aliyu, Kenneth A. Freedberg, Aima A. Ahonkhai

**Affiliations:** 1 APIN Public Health Initiatives (APIN), Abuja, Nigeria; 2 Vanderbilt Institute for Global Health, Vanderbilt University Medical Center, Nashville, Tennessee, United States of America; 3 Department of Health Policy, Vanderbilt University Medical Center, Nashville, Tennessee, United States of America; 4 Friends in Global Health, Maputo, Mozambique; 5 Institute of Public Health, Obafemi Awolowo University, Ile-Ife, Nigeria; 6 Division of Infectious Disease, Vanderbilt University Medical Center, Nashville, Tennessee, United States of America; 7 Division of Infectious Disease and General Internal Medicine, Massachusetts General Hospital, Boston, Massachusetts, United States of America; 8 Medical Practice Evaluation Center, Massachusetts General Hospital, Boston, Massachusetts, United States of America; 9 Harvard Medical School, Boston, Massachusetts, United States of America; 10 Harvard University Center for AIDS Research (CFAR), Boston, Massachusetts, United States of America; 11 Department of Health Policy and Management, Harvard T.H. Chan School of Public Health, Boston, Massachusetts, United States of America; Brigham and Women’s Hospital, UNITED STATES

## Abstract

The transition to PEPFAR 2.0 with its focus on country ownership was accompanied by substantial funding cuts. We describe the impact of this transition on HIV care in a large network of HIV clinics in Nigeria. We surveyed 30 comprehensive HIV treatment clinics to assess services supported before (October 2013-September 2014) and after (October 2014-September 2015) the PEPFAR funding policy change, the impact of these policy changes on service delivery areas, and response of clinics to the change. We compared differences in support for staffing, laboratory services, and clinical operations pre- and post-policy change using paired t-tests. We used framework analysis to assess answers to open ended questions describing responses to the policy change. Most sites (83%, n = 25) completed the survey. The majority were public (60%, n = 15) and secondary (68%, n = 17) facilities. Clinics had a median of 989 patients in care (IQR: 543–3326). All clinics continued to receive support for first and second line antiretrovirals and CD4 testing after the policy change, while no clinics received support for other routine drug monitoring labs. We found statistically significant reductions in support for viral load testing, staff employment, defaulter tracking, and prevention services (92% vs. 64%, p = 0.02; 80% vs. 20%, 100% vs. 44%, 84% vs. 16%, respectively, p<0.01 for all) after the policy change. Service delivery was hampered by interrupted laboratory services and reduced wages and staff positions leading to reduced provider morale, and compromised quality of care. Almost all sites (96%) introduced user fees to address funding shortages. Clinics in Nigeria are experiencing major challenges in providing routine HIV services as a result of PEPFAR’s policy changes. Funding cutbacks have been associated with compromised quality of care, staff shortages, and reliance on fee-based care for historically free services. Sustainable HIV services funding models are urgently needed.

## Introduction

The US President’s Emergency Plan for AIDS Relief (PEPFAR) is one of the largest commitments to date to combat a single disease globally.[1] PEPFAR has made substantial contributions toward alleviating the global burden of HIV/AIDS with targeted efforts in sub-Saharan Africa, a region that continues to bear a disproportionate share of the global HIV disease burden.[2, 3] The PEPFAR program has facilitated a rapid scale up of life-saving antiretroviral therapy (ART) over the past decade, expanding treatment to nearly 11.5 million people living with HIV in sub-Saharan Africa, compared to only 50,000 when PEPFAR was established in 2003.[1] This investment led to a 39% decline in HIV/AIDS mortality over the same period.[3]

With the inception of a new phase of PEPFAR in 2008 (PEPFAR 2.0), the program’s focus shifted from emergency response to sustainability and country ownership.[4] PEPFAR 2.0 has provided a framework for the gradual transfer of ownership of the HIV response from PEPFAR to the national governments of recipient countries.[5, 6] PEPFAR, in the course of this transition, gradually reduced its involvement in direct service delivery.[7, 8] Consequently, sub-Saharan Africa, home of the largest HIV epidemic worldwide, has experienced funding decreases exceeding 80 million USD since 2011.[9]

Following PEPFAR funding decreases, the task of developing strategies to enhance the national government’s capacity to manage sustainable national HIV programs has been met with many challenges.[10] For instance, the Republic of South Africa, the country with the largest HIV epidemic globally, has struggled to increase domestic funding enough to replace previously PEPFAR-funded providers and clinic staff lost in the transition, leading to substantial waiting, clinic overcrowding, and decreased quality of care.[8, 11, 12] With the second largest epidemic worldwide, the government of Nigeria has not only experienced fiscal challenges to support the national HIV program, but also inconsistent financial support at the state and local government levels, and low community ownership and involvement.[13] While Nigerian and South African governments both face obstacles from the reduction of PEPFAR funds, South Africa’s economy is larger and more robust than that of Nigeria, giving South Africa greater capacity to mobilize domestic funding for its HIV response.[14] In 2016, Nigeria had sub-Saharan Africa’s most poorly performing currency, with inflation of 17%.[15]

In Nigeria, PEPFAR instituted a reduction of about $83 million in yearly program support between 2011 and 2015.[15] The major areas affected by the change in funding policy were: human resources for health, laboratory services, HIV clinics operations, and logistics support (personal communication, PEPFAR Nigeria Coordinators office). These restrictions on fund utilization have raised concerns among stakeholder groups about the sustainability of service delivery.[15]

The reactions to the policy and funding changes have been mixed; some have welcomed the reduction of dependence on external funding, while others feared that the health of millions of HIV infected individuals will be jeopardized.[11, 15, 16] Our objective was to measure the impact of the PEPFAR funding policy changes instituted in October 2014 on HIV care delivery in a large network of HIV clinics in Nigeria.

## Methods

### Setting

The study was conducted at the comprehensive HIV clinics supported by APIN Public Health Initiatives (APIN) treatment network. APIN is a Nigerian non-governmental organization and a PEPFAR implementing partner (IP) through the US Centers for Disease Control and Prevention (CDC). In its role as a PEPFAR IP, APIN receives annual grants from CDC Nigeria to provide technical assistance and infrastructure support to select clinics providing HIV care and treatment service across the country.[17] APIN-supported HIV clinics provide range of services people living with HIV, for the prevention of mother-to-child transmission (PMTCT), and for orphans and vulnerable children. APIN-supported comprehensive clinics provide the most extensive services including HIV counseling and testing, antiretroviral therapy (ART) provision and laboratory services (hematology, chemistries, CD4 cell count, and viral load). Supported by a team of clinical and non-clinical staff, clinics operate as stand-alone facilities, and provide HIV care and treatment consultations two to four days a week depending on patient volume. At the time of the study, the APIN supported 30 comprehensive clinics located in three of Nigeria’s 36 states: Lagos and Oyo states in South West Nigeria and Plateau state in North Central Nigeria. With PEPFAR funding, APIN has provided care and treatment services to over 150,000 HIV-infected individuals and their families since 2004. [18]

Prior to the policy change in October 2014, the site budgets covered stipend payments to government staff working in the HIV clinics, employment of additional project staff as required to fill human resource gaps, laboratory tests for new and returning HIV patients, medications for the treatment of opportunistic infections, laboratory reagents for disease staging and monitoring of ART, maintenance of laboratory equipment, outreach programs, support for indigent patients, and staff training. After PEPFAR’s policy changes were instituted in October of 2014, overall funding was reduced to subcontract sites by 60% [personal communication, APIN finance director].

### Approach

We conducted a cross sectional study of comprehensive HIV clinics supported by PEPFAR through APIN. Clinical sites included in the study were supported with PEPFAR funding for at least 6 months both prior to and subsequent to the PEPFAR policy change in October 2014.

We collected both quantitative and qualitative data. We distributed a 75-item electronic questionnaire to clinic managers at APIN’s 30 comprehensive HIV treatment sites. The questionnaire assessed services supported before the PEPFAR policy change (October 2013-September 2014) and after the policy change (October 2014-September 2015), impact of the PEPFAR funding policy change on service delivery areas, and procedures/policies instituted by clinics in response to these changes. Questionnaires were completed by clinic management team members as a group. Surveys were reviewed for completeness and internal consistency, and one co-author (BB) conducted telephone follow-up to clarify responses when necessary.

The qualitative data were collected via open-ended questions from the distributed questionnaire, and analyzed using framework analysis.[19] The qualitative texts were read multiple times as part of the familiarization process by four members of the team (BB, CMA, OA, AAA). Discussions among authors generated two initial thematic frameworks for coding of the qualitative data, with a focus on the impact of decreased funding on clinical service delivery and coping strategies employed by HIV clinics to ensure continued delivery of quality HIV care in the context of decreased funding. Hierarchical code maps were subsequently developed to categorize data. Four code maps were developed in the framework, including: 1) poor maintenance of clinical database operations; 2) laboratory stockouts and interruptions; 3) reduced wages for staff positions; and 4) inability to subsidize clinical and laboratory services. Data analysis was conducted using MAXQDA 12 software (VERBI GmbH; Berlin, Germany).

We summarized site characteristics, including location (state), ownership model (private/not for profit, private for profit, non-profit), academic affiliation, years of HIV service availability, and number of adults in care. We compared differences in support for staffing, laboratory services, and clinical operations pre- and post- policy change using paired t-tests. Quantitative analyses were conducted in STATA 13 (College Station, TX: StataCorp LP).

### IRB approval

We obtained IRB approval from Partners HealthCare (Protocol no. 2013P000219), the National Health Research Ethics Committee (Nigeria) and Vanderbilt University Medical Center (Protocol no. 161779).

## Results

### Clinic site characteristics

Eight-three percent (n = 25) of APIN’s surveyed sites completed the questionnaire. These sites were in three of Nigeria’s 36 states; 60% of the sites (n = 15) were publicly owned, and 44% (n = 11) had academic affiliations. Of the thirty comprehensive HIV clinics supported by APIN this time, nine of the clinics (30%) were faith based, one (3.3%) was private for profit, while twenty (66.7%) were public facilities. Seven (23.3%) of the clinics were at the tertiary level of care while the others were at the secondary level. Clinics had a median of 989 adult patients in care (IQR: 543–3326). Forty percent (n = 10) of sites had provided HIV care and treatment services for more than 10 years, 52% (n = 13) for 5–10 years, and 8% (n = 2) for less than five years [Table 1].

**Table 1:** Summary of site characteristics of APIN-supported HIV treatment facilities in Nigeria.

Characteristic	N	%
**State**		
Lagos	3	(12)
Oyo	6	(24)
Plateau	16	(64)
**Ownership**		
Private for profit	1	(4)
Private not for profit	9	(36)
Public	15	(60)
**Academic Affiliation**		
No	14	(56)
Yes	11	(44)
**No. Years Providing HIV Services**		
>10 yrs	10	(40)
5–10 yrs	13	(52)
<5 yrs	2	(8)
**No Adults in Care**		
<500	6	(24)
500–4999	15	(60)
5000–9999	2	(8)
≥10000	2	(8)

N = Number % = Percent

### Impact of policy change on clinical service delivery (quantitative)

Almost all clinics (96–100%) continued to receive financial support for first- and second-line ART as well as CD4 testing after the PEPFAR funding policy change [Table 2]. However, no clinics received support for other routine drug monitoring labs (hemoglobin, alanine amino transferase, creatinine) after the policy change (p<0.001). There were significant reductions in the number of clinics receiving support for viral load testing before and after the PEPFAR policy change (92% vs. 64%, p = 0.02); staff stipends (72% vs. 8%, p = 0.01), staff employment (80% vs. 20%, p<0.001), tracking services for patients lost to follow-up (100% vs. 44%, p<0.001), and prevention/outreach services, such as community based HIV counseling and testing, and HIV service demand creation activities, (84% vs. 16%, p<0.001).

**Table 2:** Proportion of APIN clinics providing HIV clinical services before and after PEPFAR’s policy change.

Clinic Services Provided	Before PEPFAR^#^ Policy Change	After PEPFAR^#^ Policy Change	p-value
	N (%)	N (%)	
First Line ART*	25 (100)	25 (100)	0.88
Second Line ART*	24 (96)	25 (100)	0.58
CD4 Count	25 (100)	25 (100)	0.33
HIV RNA	23 (92)	6 (64)	0.02
Monitoring Labs^+^	25 (100)	0 (0)	<0.01
Staff Stipend	18 (72)	2 (8)	0.01
Staff Hiring	20 (80)	5 (20)	<0.01
Patient Tracking	25 (100)	11 (44)	<0.01
Outreach Services	21 (84)	4 (16)	<0.01
Staff Training	24 (96)	5 (20)	<0.01
Generator Fuel	25 (100)	7 (28)	<0.01
Information Technology Support	24 (94)	10 (40)	<0.01

*Antiretroviral Therapy

^+^Hemoglobin/Alanine Aminotransferase/Creatinine

^#^ President’s Emergency Plan for AIDS Relief

### Impact of policy change on clinical service delivery (qualitative)

**Fig 1:**
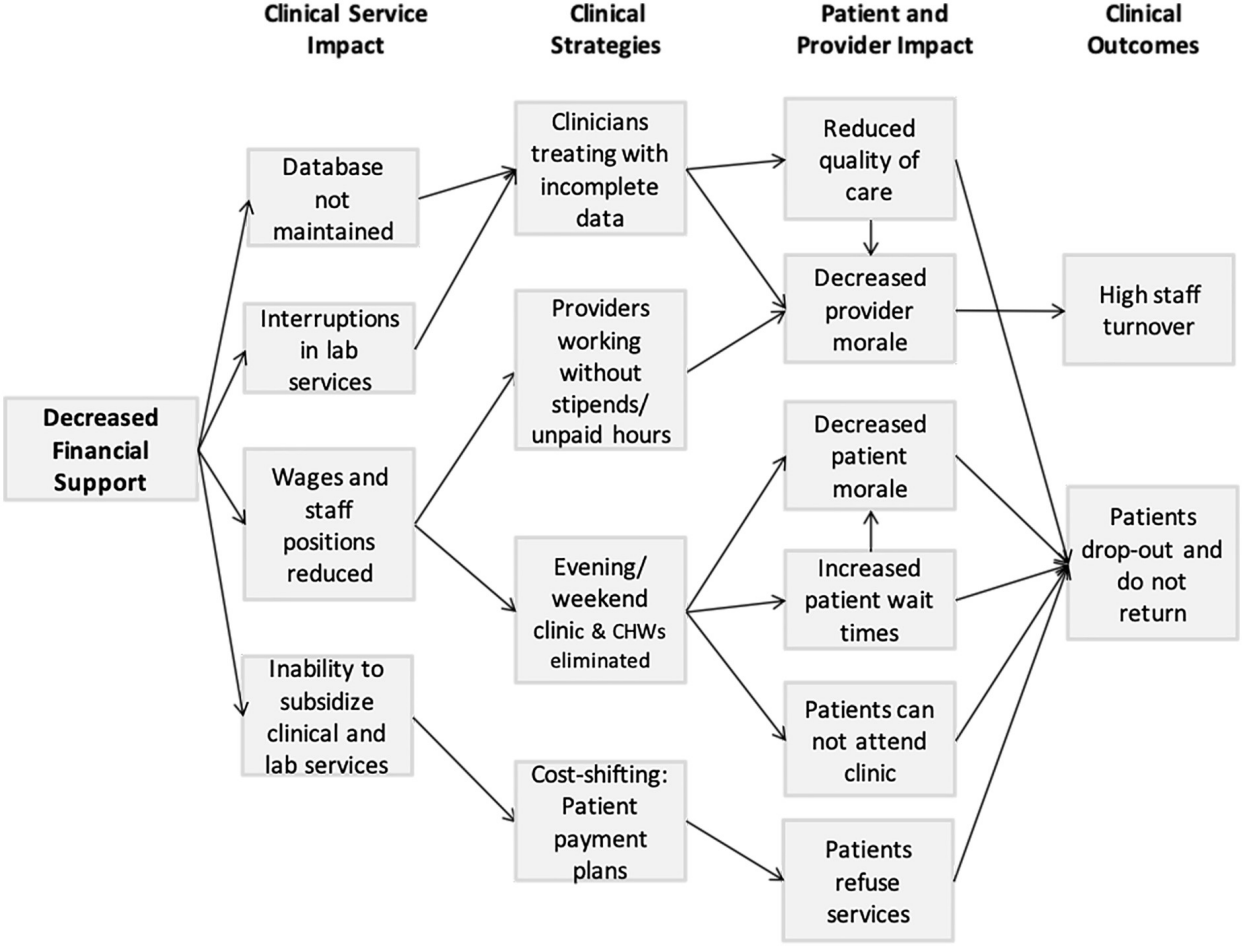
Thematic analysis of impact of PEPFAR policy changes on HIV clinical services and programmatic responses in APIN clinics.

Thematic analysis revealed that the reduction of PEPFAR funds impacted clinical service delivery via: (1) decreased maintenance of clinical database operations; (2) interrupted laboratory services; (3) reduced wages and staff positions; and (4) unsubsidized critical clinical and laboratory services (Fig 1). At many clinics, these ramifications resulted in the inability to maintain clinical databases due to equipment failure, personnel reductions, and limited internet access. Clinicians often made clinical decisions based on incomplete laboratory data due either to poor data entry, backlog in lab processing, or inability or unwillingness of patients to pay for and undergo laboratory testing. Thus, unintentionally, PEPFAR funding reductions on APIN clinics created an environment in which the quality of care provided was challenged.

### Strategies adopted to maintain clinical services

Clinic managers described instituting one or more of five coping mechanisms to address changes resulting from PEPFAR policy. Almost all sites (96%) introduced user fees to cover the costs of: lab monitoring tests (96%), hospital registration (32%), or clinical consultation (20%); yearly median fees were $40 USD (IQR:$21-$69 USD) and ranged from $4 to $166 USD. Some sites addressed funding needs by focusing on external fundraising efforts (16%). Others (20%) instituted task -shifting to overcome human resource constraints, and others (20%) increased the use of volunteer staff. Few clinics (12%) limited the number of lab testing requests that could be processed [Table 3].

**Table 3:** Coping mechanisms adopted by APIN supported facilities in response to policy changes.

Mechanism	N (%)
User Fees	24 (96)
Task shifting and multitasking	5 (20)
Increased use of volunteers	5 (20)
Sourcing for funds externally	4 (16)
Limiting lab test requests	3 (12)

All but one APIN clinic (n = 24, 96%) shifted lab costs to patients, but some clinics exempted vulnerable patient groups, such as pregnant women (15%), children (42%), and indigent patients (39%) from these fees. Other strategies adopted to cushion the effect of user fees included direct involvement of patient advocates in setting the fee schema, installment payment plans, and decreasing the required number of clinic visits. These strategies were primarily described by private and public, tertiary clinics.

Although PEPFAR support remained for CD4 cell count testing, the lack of support for laboratory monitoring tests (Creatinine, ALT, and Hemoglobin) created a ripple effect in many clinics. Patients who did not pay for laboratory monitoring deprived the labs of revenue needed for basic operations; and while CD4 and, in some cases, viral load testing were still supported by PEPFAR, some labs did not have the capacity to do these tests. Additionally, the capacity for laboratories to log samples and transfer to reference labs was constrained by reduced funding for logistics support.

One clinic site reported:

“The patient pays out of pocket and many complain about cost; the lack of money cause[d] some [patients] to skip the tests.”

(Public, secondary hospital)

In response to the PEPFAR policy change, APIN supported clinics eliminated several services that optimized patient convenience, including evening and weekend clinical appointments, and daily laboratory hours. Clinicians asserted that these changes reduced the quality of clinical care provided. Clinicians suggested that patient satisfaction was also impacted, as patients had to endure lengthy waiting times to receive ART. Also, with less fewer resources to support outreach services like home-based care, patients could not receive effective community-based services to support their care needs. At one health facility, a clinician reported:

“The medical team is markedly stressed and [patient] waiting time has increased. Patient dissatisfaction has increased. Non-scheduled visits have reduced as waiting time is so long. Early diagnosis of OI (opportunistic infections) and associated HIV diseases has reduced markedly as patients take their complaints to non-HIV trained health facilities or CAM (complementary or alternative medicine) outlets.”

(public, tertiary hospital)

### Impact on providers

Providers were also negatively impacted by funding reductions. Employees reported reduced wages, and elimination of essential full time HIV clinical positions (government employees who received additional stipends for working in HIV clinics). In the absence of receiving these additional stipends, former “full time” clinical staff were unwilling to take on what they considered to be additional work, causing delays in clinic wait times. According to one of the clinic managers:

Because (employment of additional project staff) has stopped, we currently have fewer staff attending to our clients with the resultant work over load”

–(Public, tertiary hospital)

Changes in staffing structure also led to high turnover and decreased provider morale. At one facility, it was reported that:

“…staff attrition from the hospital is very high. We get new staff almost monthly and we have no funds to train them locally. This affects the quality of services provided to the patients.”

(Faith-based, secondary hospital)

## Discussion

Transitioning the responsibility for all or parts of a donor funded program to host government or local stakeholders is common in development programs, and is usually considered to be a sustainability strategy.[2, 10, 20] Indeed, development programs that do not plan to successfully incorporate a blueprint for local country ownership may be criticized for facilitating donor dependency.[16] Despite the importance of the successful transition of PEPFAR efforts to donor recipient countries, the nature of these transitions is complex, and, has been met with challenges.[8, 11] With increasingly aggressive global targets such as the UNAIDS 90-90-90 goals, many have feared PEPFAR supported programs would not only fall short of these targets, but also lose the tremendous gains established in the earlier PEPFAR era.[21] Our analysis highlights that clinics in Nigeria’s APIN network have had major challenges in the provision of HIV services in the wake of the PEPFAR funding policy change. Providers describe reductions in the quality of care administered and in clinic attendance, along with human resource gaps as the greatest threats to delivering effective HIV care.

There are several defining elements of high quality HIV care, but appropriate and timely provision of ART is a central component.[22, 23] Prompt ART initiation can prevent excess mortality among infected patients and prevent HIV transmission to uninfected individuals.[24, 25] After the PEPFAR policy change, funding for first and second line ART remained for APIN clinics, but other equally critical inputs for effective ART administration were compromised. Clinicians described difficulty obtaining laboratory results to determine eligibility for and response to ART. Although PEPFAR still provided funds for CD4 testing, viral load testing was no longer supported in many facilities. Critical laboratory infrastructure was undermined because many patients did not pay for required laboratory tests that were no longer supported by PEPFAR, but helped to subsidize laboratory operations. The downstream impact of these changes, included delays in ART initiation, delays in identifying treatment related toxicities, and reliance on clinical criteria for HIV treatment failures—a practice which has proven inferior to viral load and CD4 testing.[26, 27] All of these downstream effects have major potential for worsening clinical outcomes.

Charging user fees for patients was the single most common response adopted to overcome financial shortages after PEPFAR cutbacks. User fees were instituted by all but one of the surveyed clinics. Early in the global AIDS response, many patients were required to pay out-of-pocket fees for ART, clinical consultation, and transportation.[28–30] During this period in Nigeria, one study showed that 56% of annual household income for homes with an HIV-infected family member was taken up by healthcare expenses and lost income [31]. Meta-analyses of low and middle-income countries in this early scale-up period also reported that user fees may decrease care utilization and healthcare quality. Clinics with user fees had a 30% reduction in the proportion of patients achieving virologic suppression and 4-fold increased risk of attrition and death compared to those without fees [29, 30, 32]. Data such as these motivated PEPFAR and other key stakeholders to make HIV treatment available free of charge to many persons living with HIV and AIDS in low and middle income countries (LMIC).[2]

Reduced funding for outreach services, laboratory monitoring, and reduced staffing, may have further compounded the deleterious effects of user fees, further destabilizing care delivery systems. Ironically, while most APIN-supported clinics called on user fees to fill a monetary gap and ensure ongoing service provision, a growing literature has focused on the role of financial incentives to improve HIV-related health outcomes in low resource settings.[33–35] APIN-supported clinics employed several strategies to cushion the effect of user fees on clients, including exemption of fees for vulnerable patient groups, offering of installment payment options, and collection of donations to facilitate cost sharing. However, the distribution of these strategies was skewed towards private clinics and public tertiary clinics. In contrast, public secondary facilities, which made up 44% of surveyed clinics, did not have as much autonomy to provide such safety nets. This situation suggests there may be an inequitable distribution of user fee burden on patients served by APIN clinics.

Human resource shortages have been a persistent challenge during the rapid scale-up of ART in LMICs.[36] PEPFAR-supported sites have used creative strategies such as use of retired staff, formal and informal task-shifting, and development of new cadres of health workers to meet human resource needs for HIV care.[36] In Nigeria, HIV program staff were incentivized with stipends and technical training. With reductions in PEPFAR funding, such stipends were no longer available, along with a level of security that provided some cushion from often delayed government salaries. APIN-supported clinics reported drastic staffing shortages, and increased workloads placed on the staff that were retained. The combination of an under-resourced working environment along with frustrated and unhappy patients has led to high turnover among retained staff.

Important lessons learned from other donor transitions suggest that identification of alternative funding sources is a critical element for successful transition.[10, 20, 37, 38] Several innovative funding mechanisms have been identified to support domestic HIV programming in resource-limited settings such as tax/levy programs, debt buy-downs, and community-based insurance; but few have been successfully operationalized.[39] One notable example is Zimbabwe’s levy program which taxed formal sector income, to raise $85 million USD in revenue for its national HIV program between 2008 and 2012.[39] APIN’s experience in Nigeria highlights many downstream effects caused by the gap left between PEPFAR funding and national support when alternative funding is not successfully sourced.[10] These effects have negatively impacted the health system, providers, and patients with human resource shortages and compromised quality of care. With user fees instituted by most clinics as the primary response to financial shortage, most clinics reported underutilization of clinical services and major potential for worsening of clinical outcomes. As Nigeria and other PEPFAR supported countries continue to navigate donor transitions, stakeholders must remain focused on implementing affordable and successful models for sustainable service delivery.

## Supporting information

S1 Dataset Health system coping mechanisms in response to PEPFAR policy change. (XLSX)
